# Combining physical and virtual contexts through augmented reality: design and evaluation of a prototype using a drug box as a marker for antibiotic training

**DOI:** 10.7717/peerj.697

**Published:** 2014-12-16

**Authors:** Sokratis Nifakos, Tanja Tomson, Nabil Zary

**Affiliations:** Department of Learning Informatics Management and Ethics, Karolinska Institutet, Stockholm, Sweden

**Keywords:** Antimicrobial resistance, Augmented reality, Antibiotics, Mobile learning

## Abstract

**Introduction.** Antimicrobial resistance is a global health issue. Studies have shown that improved antibiotic prescription education among healthcare professionals reduces mistakes during the antibiotic prescription process. The aim of this study was to investigate novel educational approaches that through the use of Augmented Reality technology could make use of the real physical context and thereby enrich the educational process of antibiotics prescription. The objective is to investigate which type of information related to antibiotics could be used in an augmented reality application for antibiotics education.

**Methods.** This study followed the Design-Based Research Methodology composed of the following main steps: problem analysis, investigation of information that should be visualized for the training session, and finally the involvement of the end users the development and evaluation processes of the prototype.

**Results.** Two of the most important aspects in the antibiotic prescription process, to represent in an augmented reality application, are the antibiotic guidelines and the side effects. Moreover, this study showed how this information could be visualized from a mobile device using an Augmented Reality scanner and antibiotic drug boxes as markers.

**Discussion.** In this study we investigated the usage of objects from a real physical context such as drug boxes and how they could be used as educational resources. The logical next steps are to examine how this approach of combining physical and virtual contexts through Augmented Reality applications could contribute to the improvement of competencies among healthcare professionals and its impact on the decrease of antibiotics resistance.

## Introduction

### Antibiotic resistance is a global health challenge

The widespread inappropriate use of antibiotics provokes the manifestation of antibiotic resistance organisms. Antimicrobial resistance (AMR) is one of the biggest public health challenges (Tan, 2008). The effectiveness of antibiotics is decreasing and resistance to antimicrobial therapies is rising, thereby leading to an increase in morbidity, mortality and health care expenditure (Coast, Smith & Miller, 1996). In particular, while globalization plays a key role in increasing the vulnerability of all of the countries around the world, resistance remains a global public health threat, and individual actions cannot protect the health of its population against it (Smith & Coast, 2002). While examining the causes of antibiotic resistance, a complex and insufficient mechanism can be observed, which includes human behavior and the different levels of society. As a result, the consequences affect everybody in the world (Smith & Coast, 2002). We could possibly refer to some similarities of this phenomenon with climate change. Until now, a significant amount of research has been conducted in order to describe the different facets of antibiotic resistance and to document the interventions needed to meet the challenge, even though a large scale of coordinated actions is absent (Coast, Smith & Miller, 1996). It is a common assumption that without antibiotics, a list of achievements in modern medicine, for instance major surgery, treatment of preterm babies, and cancer chemotherapy, could not exist without an effective treatment for bacteria infection. It could be argued that in a few years we might be faced with dire setbacks on many levels and in many areas: medically, socially and financially (Laxminarayan et al., 2013).

### Educational strategies

There are efforts being made widely around the world between cultures and regions on multifaceted and multilevel interventions that defy local barriers and beliefs. Recently reviewed educational strategies indicate the essential role of education for health-care workers, laboratory staff and the public in appropriate antibiotic use and antimicrobial resistance (Pulcini & Gyssens, 2013). Defining the complexity of the antimicrobial resistance phenomenon, education alone might not be a powerful enough intervention but it could generate knowledge, which could be essential for health-care professionals in understanding and contending with the resistance control systems (Pulcini & Gyssens, 2013). In order to puzzle out this complex problem, information needs to be clarified by policy makers about antibiotics and their effect on public health (Laxminarayan & Klugman, 2011). Drug Resistance Index Social (DRIS) education and awareness campaigns for the public population could also possibly generate an understanding that can support the prescriber withhold antibiotics (Huttner et al., 2010). It has been observed that these campaigns could contribute to more careful use of antibiotics.

### Augmented reality

Augmented Reality (AR) is a technology that provides the opportunity for computer-generated virtual imagery information to be overlaid onto a direct or indirect live real world environment in real time (Azuma, 1997). AR bridges the gap between the real and the virtual in a seamless way (Chang, Morreale & Medicherla, 2010). Moreover, Augmented Reality complements the real environment and does not replace it as virtual reality applications do (Azuma, 1997).

However, AR applications are not yet being used on a large scale in the educational system (Yuen, Yaoyuneyong & Johnson, 2011). Although Augmented Reality is not new, its the dynamics in the field of education have only been explored recently. Unlike other technologies in computer science, augmented reality interfaces provide the user with interaction features between the real and the virtual world, a tangible interface transformation, and tools for the transition from the real to the virtual world (Ong et al., 2011). It is, however, the responsibility of teachers to cooperate with researchers in this field in order to explore how the features of augmented reality can be implemented in the best way in an educational environment (Cuendet et al., 2013). These applications could be used through mobile devices, but it would be more practical and effective to be implement the use of digital scanners in order to promote the collective process and to set up a direct communication between instructors and trainees (Squire & Klopfer, 2007). Nevertheless, the dynamic characteristics of Augmented Reality technology should be carefully analyzed in order for its transition as an educational tool to be feasible.

In the 2010 and 2011 Horizon reports, augmented reality has been mentioned as a promising technology for education. According to Kamphuis et al. (2014) augmented reality is a promising educational tool for delivering meaningful learning. Moreover, it should be mentioned that AR technology provides also organizational advantages such as (i) a training environment that is almost the same as the professional work environment, (ii) collaboration between users will support authentic learning, (iii) real time interactive feature of AR provides immediate feedback to the user, (iv) experts or instructors are not always necessary to observe trainees performance, (v) situated learning: “Just in time” and “Just in place”.

A number of studies were found for augmented reality in medical education and more specifically AR training systems for medical learning tasks such as visualizing parts of human body and laparoscopy training session with augmented reality. These studies are attempting to explore the dynamics of augmented reality for complex learning domains in medical education (Azuma, 1997).

Another study from Zhu et al. (2014) performed an integrative review for augmented reality in healthcare education. The results from this study indicate the acceptance of AR systems as learning technology tools in healthcare education. There is no empirical study to support this claim and to show how exactly augmented reality improves effectively the training skills of the trainees.

### Aim

Antibiotics have been considered a determining factor for saving lives and minimizing the suffering of patients for more than sixty years. The widespread inappropriate use of antibiotics provokes the manifestation of antibiotic resistance organisms. Antimicrobial resistance (AMR) is a major public health challenge (Rammanan et al., 2013). Previous studies (Munck et al., 1999; Butler et al., 2012; Welschen et al., 2004; Flottorp et al., 2002) have shown that combining educational methods and intervention strategies for general practitioners can reduce antibiotics prescriptions by 3% to 12% (Welschen et al., 2004; Flottorp et al., 2002). Developing effective educational methods to teach healthcare workers could therefore further reduce unnecessary prescriptions which lead to prescription errors and are considered to be one of the critical factors for the antimicrobial resistance global health issue. Educational technology interventions can support the decrease of prescription errors among healthcare professionals.

Therefore the aim of this study is to investigate how the visualization of information in medication boxes from an AR scanner may support antibiotics prescription education.

## Method

A design-based research approach was applied, and consisted of four connected phases (Reeves, 2006):

• Analysis
• Development of solutions
• Iterative cycles of testing
• Production of design principles

The approach of this study is based on the principles and the basic structure of building applications for educational purposes. This research approach is being used widely in education since it investigates the innovation with the usage of technology-based initiatives because according to Kelly (2004) it “embraces the complexity of learning and teaching and adopts interventionist and iterative posture toward it.” According to Reeves & Hedberg (2003), some of the key elements include addressing complex problems in collaboration with practitioners, integrating design principles with new technologies to develop practical solutions to the problem and conducting effective evaluations to refine the proposed solution and identify new design principles.

### Analysis

According to Herrington, Reeves & Oliver (2010) the analysis phase addresses three key areas: the detection of the problem, the literature review and the practitioner’s experience. The targeted goal in the analysis phase is to identify the problem and investigate what has already been done in the same or related fields.

The problem as it has been set in the introduction is antimicrobial resistance. Since this global health issue is very complex, combining several scientific and social areas, this project focused on the educational structures governing this area. Different explorative methods had been used for mapping the educational field of antibiotics. For this purpose a web survey consisting of 15 questions was used in this study. This web survey was not based on any specific standard of surveys since it was the first phase of approaching the researched area, and the goals were to explore information regarding the antibiotic educational process, the prescription process in hospitals, the private prescription process, possible training sessions for antibiotic education, and courses in medical schools which are important for the participants for antibiotic knowledge. Eight resident doctors and two registrar doctors participated in this study. All of them were working within the Stockholm area in public hospitals. The recruitment was based on an open announcement in Södertälje hospital in Stockholm, Sweden. With this survey, the practitioner’s experience regarding the educational experiences they had in antibiotics as well as the tools that they currently use when they prescribe antibiotics were explored. Since the design research approach focuses on the knowledge of the practitioners and seeks to use their insights on the research and design and thereby providing potential solutions to the educational process, the collection of these data was critical for this study.

In parallel with the web survey, a literature review was conducted in order to explore information regarding the educational area of antibiotics, as well as to examine which technology could possibly be useful for supporting the educational process and enriching the educational experiences. This process also supported the study by providing information from other research in similar areas. Moreover, the literature review supported the exploration of research in technological educational tools. More specifically, it focused on researchers whose main academic interests are mobile educational tools and augmented reality in the field of education.

For the literature review, different databases have been used according to different science fields. The Karolinska Institutet’s e-library databases (PubMed, Web of Science, CINAHL) have been used for medical and healthcare material. More specifically, research in the field of antimicrobial resistance, antibiotics, antibiotic education, public health, educational tools in antibiotic education, antibiotic guidelines, antibiotic policies, virtual patients in antibiotic education, clinical pharmacology. For the technological material, Stockholm’s University e-library databases have been used (IEEE, Scopus). A significant number of papers were found in the fields of mobile, mobile educational tools, augmented reality, mixed reality, augmented reality and education, mixed reality and education, augmented reality integration, augmented reality and data collection, contextual learning and ubiquitous learning.

### Development of solutions

Following the study of Herrington, Reeves & Oliver (2010), a more targeted literature review was conducted together with relevant theories, existing frameworks and design principles. The solution process started with the development of an Augmented Reality prototype and was based, at the beginning of the process, on assumptions of how this prototype will function and its main features. After this, different Augmented Reality platforms were tested, focusing more on the prototype development aspects and based on the literature review.

### Iterative cycles of testing

**Figure 1:**
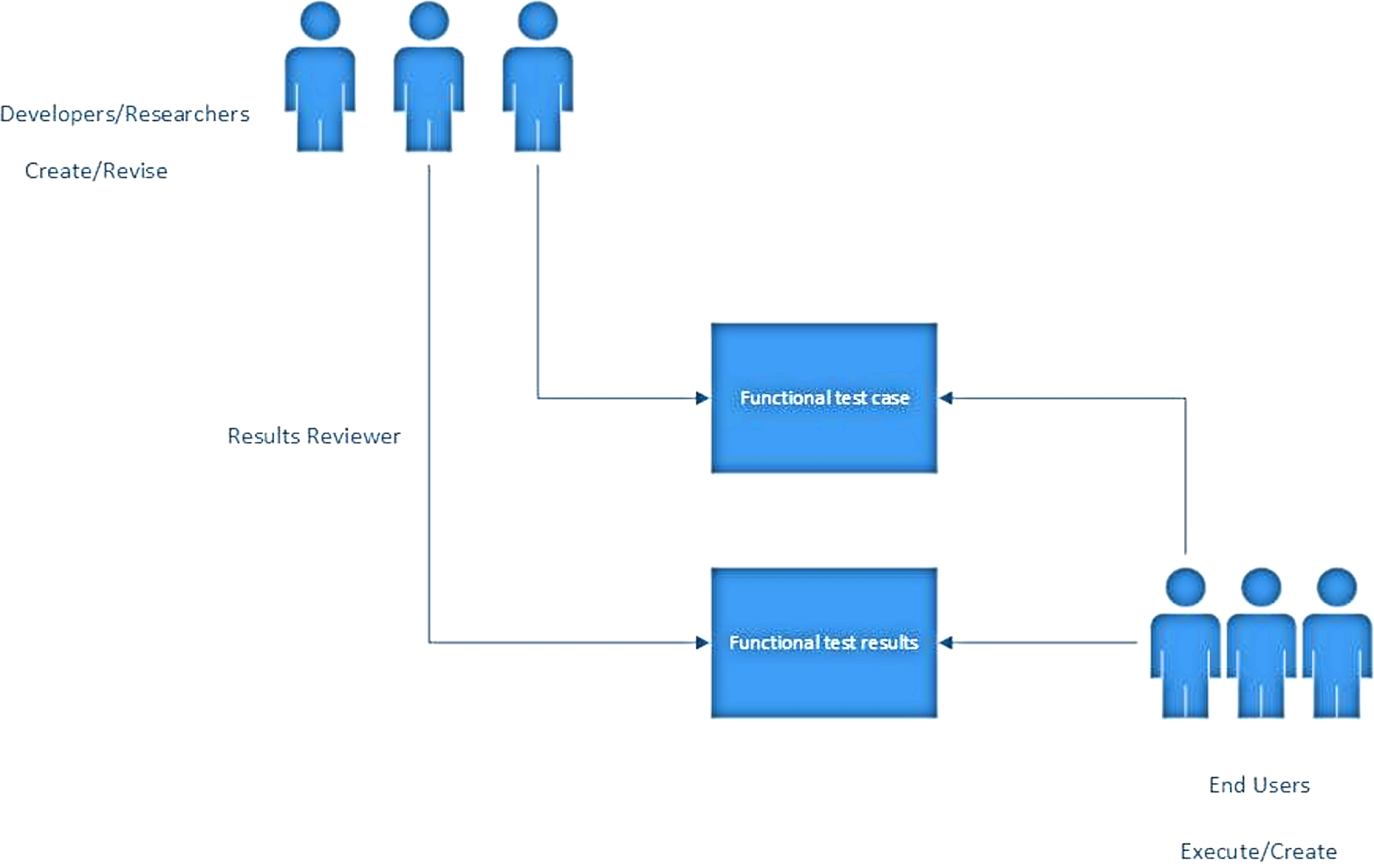
Functional test case diagram.

A “think aloud” session was organized and included eight final-year undergraduate medical students from the Karolinska Institutet. The recruitment of the students was based on an open announcement. The selection criteria of this group of students were based on the fact that the designed AR prototype will be used by medical students, and the fact that the testing prototype requires that the user have a basic knowledge of antibiotics in order to understand the prototype’s principles and dynamics (Fig. 1).

Thinking-aloud tests conformed to the traditional Ericsson & Simon (1996) (E&S) model. The process proceeded within the context of designing an AR prototype for antibiotic education. Before the beginning of the “thinking-aloud” session, a simple AR prototype was developed based on the information collected from the literature review.

The prototype was tested in terms of functionality and viability. A functional test plan was conducted with the usage of test cases. The test cases can reveal flaws in the functional specs. The users were asked to use their phones and install an AR scanner. In parallel, we ran a test case based on a training session for antibiotic prescription.

### Production of design principles

The prototype was built on the idea that the antibiotic medication box could be useful for the antibiotic educational process since it is used in hospitals following the completion of their studies. One antibiotic box was used for this prototype (Kåvepenin) visualizing additional information when the box was scanned by a mobile device. More specifically, the Kåvepenin box was chosen as a marker. This prototype was the backbone upon which we based the “thinking-aloud” session in order to facilitate the students in understanding how AR works and what its main features are. At the beginning of the session, a short presentation took place, during which videos and images were used to show students the AR technology in the medical field as well as other fields.

Following this, we started discussing the potential usage of AR. The discussion started by setting the context in which the students assumed that this prototype could fit better. The potential of using real antibiotic products and the features that AR could provide in this design were examined. Moreover, we examined the contextual framework within which the AR technology could be incorporated and designed. Whenever a discussion was loaded from information a short presentation on the projector showcased how the prototype would look. This fact triggered the beginning of new ideas regarding the prototype’s User Interface and its different functions. Notes were kept from their observations and proposals, and the conversation moved forward in this manner. For analyzing the data of the web-based questionnaire, thematic context analysis was used. This technique structures the collective answers by dividing them into categories of identified themes.

## Results

From the collected information, it was concluded that physicians in a real clinical setting follow a routine when they prescribe an antibiotic to a patient. However, the process is complex and in some cases different prescription methods are used. This study explores the most common habits in the prescription process; it does not investigate individual patients’ incidents.

Nine out of ten resident doctors answered that the two most important elements in the prescription process are the guidelines and the side effects. Nine out of ten resident doctors also mentioned that they use Strama (paper) as a guideline consulting tool and six out of ten use the “wise list” (Kloka Listan) (http://www.janusinfo.se/In-English/) for the same purpose (Table 1).

**Table 1:** The physicians’ opinions on what aspects of antibiotics are important during the prescription process.

**Important information during the** **prescription process in hospital**		**Tools that the doctors using** **during a prescription process**	
Antibiotic guidelines	90%	Strama (paper based)	90%
Antibiotic side effects	90%	Strama (mobile)	10%
Clinical manifestation	10%	Antibiotic list	60%
Allergies	10%		
Earlier, failed antibiotic treatment	10%		

The information that was collected is analyzed below in order to extract useful information for the prototype.

### Antibiotic guidelines

According to the British Infection Association and Health Protection Agency, guidelines are “intended to aid selection of an appropriate antibiotic for typical patients with infections commonly seen in general practice. Individual patient circumstances and local resistance patterns may alter treatment choices”.

### Antibiotic side effects

According to the University of Michigan Health System “Common side-effects include diarrhea, resulting from disruption of the species composition in the intestinal flora, resulting, for example, in overgrowth of pathogenic bacteria, such as clostridium difficile”.

### Strama

According to the Swedish Strategic Programme against Antibiotic Resistance, Strama is “an advisory body with the remit to assist the Swedish Institute for Infectious Disease Control:

(1) Matters regarding antibiotic use and containment of antibiotic resistance.
(2) Facilitates an interdisciplinary and locally approved working model, ensuring involvement by all relevant stakeholders including national and local authorities and non-profit organizations” (http://en.strama.se/dyn//,85,3,78.html).

### Literature review

In the literature review that was performed, with the aim to explore studies in the similar field with the current study, the following results were retrieved (Table 2):

**Table 2:** Literature review results with the usage of specified terminology.

Topic	Total papers	Examined
Augmented reality	183,691	124
Augmented reality in healthcare	3,280	23
Augmented reality and medical education	2,833	26
Augmented reality and antibiotics	6	6
Design-based research	23	11
Information technology and medical education	182	27
Antibiotics and prescription processes	11	11

Augmented reality plays a significant role in technological research. Since this technology can be applied in many different areas, there were a wide range of research papers. Focusing more in the medical field, the extent of the research papers increased but was still too general since the medical sector covers many different departments, from surgeries and diagnostics to practical training and inter-professional skills. From the final group of searched papers, 118 papers were chosen for analysis and reviewing, in order to investigate the methods that different researchers followed in the development of AR prototypes for educational purposes.

From the examined studies in Augmented Reality technology and Medical Education, it was concluded that AR is implemented in some areas of medical education, for instance in dermatology courses, surgery courses and in some microbiology and biology courses. However, no research was found in the field of antibiotic prescribing education.

### Prototype development

**Figure 2:**
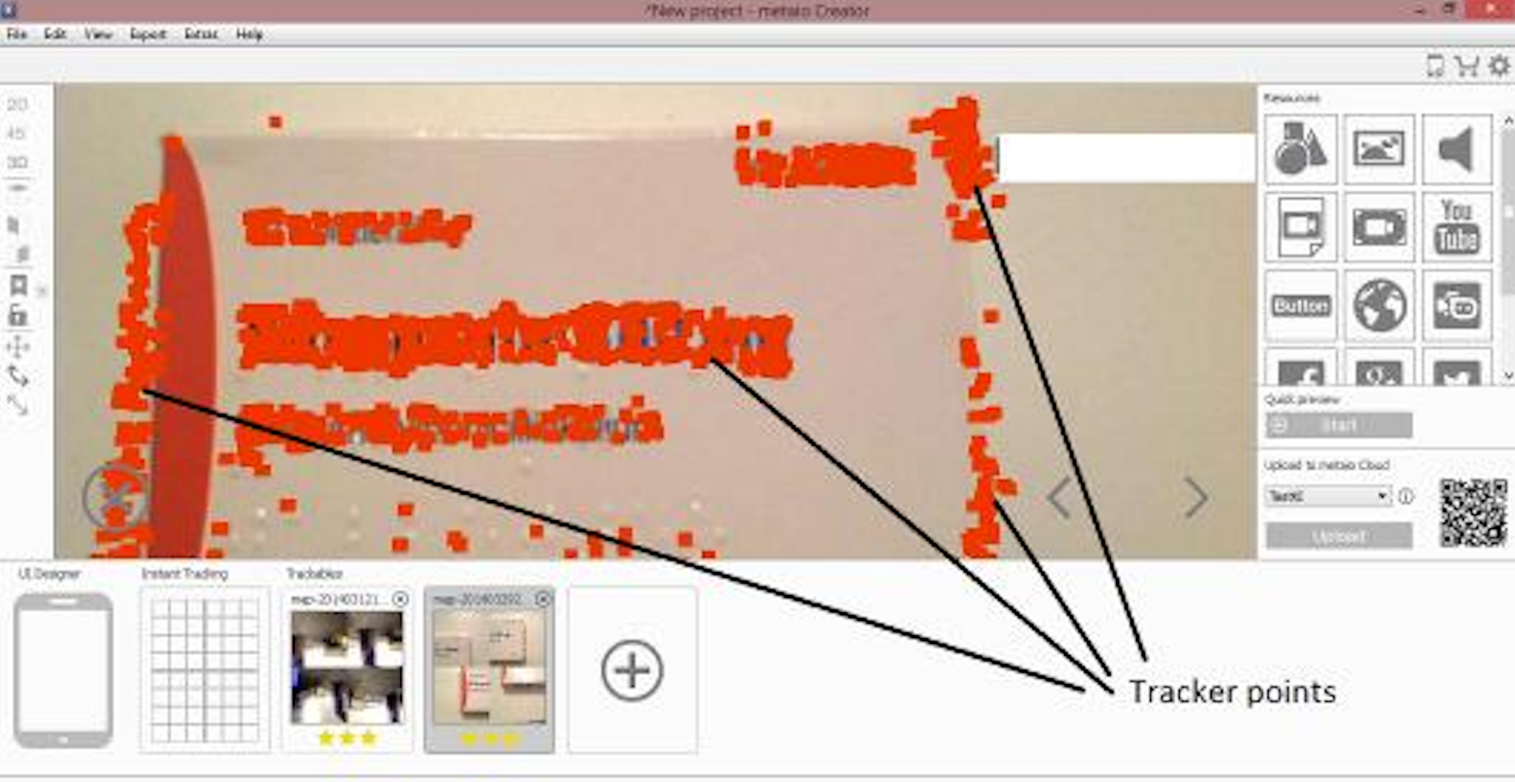
The development process of an AR traceable object.

The first pilot prototype was designed and developed based on the first open web survey. In Fig. 2, the development platform during the prototype development is shown.

The tracker points are used by the computer system in order to build a traceable object which will be recognizable from the system. This will be used as an augmented reality scanner. The 3D map of the object is then uploaded to a channel, which is simply a short space provided by the development platform in order to upload projects. The server automatically generates a QR code which is the key for accessing the channel. The user scans the QR code and inserts it into the channel. Next, the user scans the real object and the augmented information which has been saved in the 3D map is visualized in the user’s mobile device.

### Objects design

For the objects’ development SketchUp 3D was used. We designed simple 3D clickable buttons with name categories:

• Button 1: Antibiotic Guidelines
• Button 2: Antibiotic proper usage
• Button 3: Run a test

**Figure 3:**
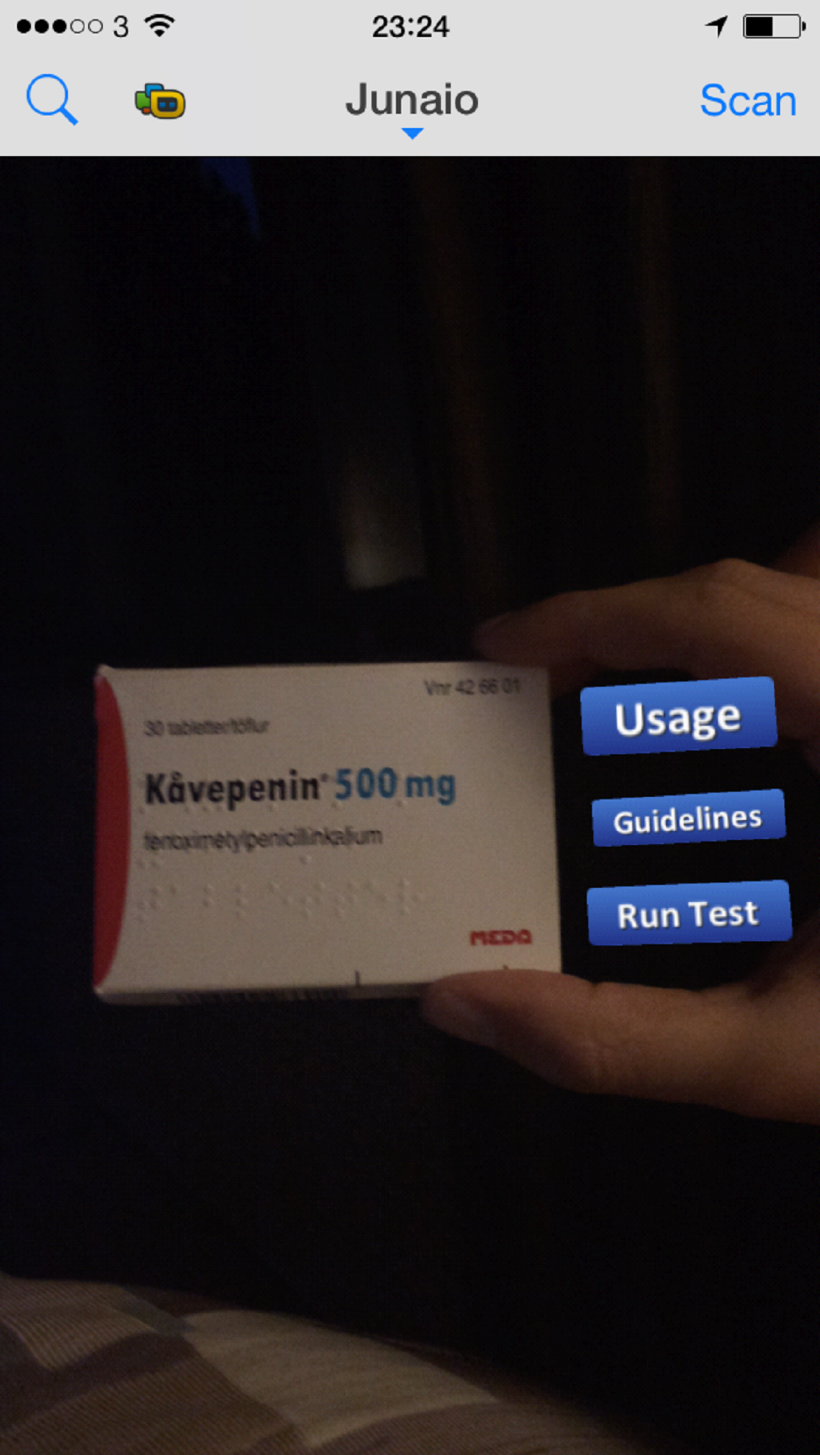
Presents the user’s display when the drug box is scanned by an AR scanner.

We made two functional buttons, named “forward” and “back.” We designed a transparent text presenting the information according to the title that was written in the button. For instance, for Button 1, information for Antibiotic Guidelines were written. This model was imported in the AR platform as “3D button”. When the user scans the antibiotics (Kåvepenin) drug box the buttons appeared in the right side of the box as it is presented in the following figure. By clicking one of the buttons, the information appearing in the user’s display (Fig. 3).

### Evaluation of the prototype

The prototype was evaluated by the end users (students) in terms of functionality and viability. It was observed that the end users were not familiar with the augmented reality technology and its functions. The functionality test, though, shows a high level of acceptance of AR technology as a training tool. The users were satisfied with the combination of a real object (drug box) which they will use in the real context in the future, with the digital information. The process was also very fast without the need of the users to use special equipment, log in information or special training to use the application.

On the other hand, some observations regarding the user interface and the functionality of the application from mobile devices should be mentioned. Some of the users found it difficult to use their mobile phones and interact with display information.

Some of the student’s quotes considered for evaluating the drug box prototype are presented below:

Participant 6:

“*It seems useless to have a training session with the drug box…for me the interface is quite difficult to use it*”

Participant 8

“*The drug boxes are changing every six months approximately…probably we need to keep only the brand name as a tracker and not the whole drug box”*

Participant 2:

“*It would be useful to have a picture that refers to antibiotics and with the usage of AR it takes real life*”

Participant 5:

“*I want also in this prototype to have a video for informing the students about the causes of non-proper prescription and the antimicrobial resistance*”

Participant 6:

“*I would like to have pictures or 3D objects of current active antibiotics that the hospitals are using in Sweden and to visualize the basic guidelines of each agent*”.

## Discussion

Antimicrobial resistance is currently one of the biggest global health threats in the world. This problem is complex and incorporates many different scientific fields. This study approached the problem from the educational perspective and attempted to answer the scientific questions that were set in order to examine the potentiality of modern technologies, such as Augmented Reality, to be applied as an educational tool in the education of antibiotics. The research question to identify the aspect that are important during a prescription process was answered. This information seeded the study in terms of prototype development.

### The prototype development

For the development of this prototype we used the Metaio Platform Beta Version. On the one hand, in this version the developer is flexible enough to develop an AR prototype in limited time. The platform also provides the opportunity to upload the prototype and test it in real time. On the other hand, in this version the development features are limited. The developer is confined to using the specific tools that the platform provides. Future studies might consider more flexible platforms such as Unity Vuforia for their prototype development.

Since we know from our research what information of antibiotics can be used in antibiotic prescription training session, it is a matter of design how this information will be designed and set for AR visualization. For the drug box prototype we used 2D clickable buttons as visualized objects. We chose this structure in order to separate the different information categories.

Approaching the antibiotics field from the training perspective, this study shows which information on antibiotics is important during a prescription process in a hospital. Based on this information, we developed an augmented reality prototype which aims to support the training of antibiotic prescription education. The results indicates that augmented reality technology had a high level of acceptance among the medical students who participated in this study, but the final prototype needs further improvement.

### Limitations of the study

This study investigated the design process of an AR prototype for supporting education about antibiotic prescription. The Design Research methodology was followed and the structure was based on its principles. This study didn’t complete more than one cycle of testing for the developed prototype. This fact should be taken under consideration. Going deeper in the methodology different methods could possibly be implemented, such as observation in real context and the examination of educational processes for antibiotics. Another possible approach would be to involve teachers in the development process and take their insights under consideration. As an alternative, the model methodology was examined: The basic principle of this methodology is “the purposeful abstraction of a real or a planned system with the objective of reducing it to a limited, but representative, set of components and interactions that allow the qualitative and quantitative description of its properties.” This alternative should be examined by the future researchers, especially by computer scientists, in terms of AR model development and AR model in medical education.

## Conclusion

Our results show how AR technology can be used to support a training session for the antibiotic prescription process and open the way for future research by using real objects as educational resources. It also illustrates (http://www.janusinfo.se/In-English/) the dynamics of mobile technology and how it could be used for enriching the educational process in different areas by using real objects in real time without requiring physical presence in a study place. These dynamics need further exploration in order to identify ways to increase the interactivity, understandability and reduce complexity in medical education with mobile technology.
